# Implementation of Web-Based Respondent-Driven Sampling to Recruit Users of Electronic Nicotine Delivery Systems in Brazil: Cross-Sectional Survey

**DOI:** 10.2196/81573

**Published:** 2026-01-27

**Authors:** Neilane Bertoni, Andre Salem Szklo, Francisco Inacio Bastos

**Affiliations:** 1 Brazilian National Cancer Institute (INCA) Rio de Janeiro Brazil; 2 Oswaldo Cruz Foundation Rio de Janeiro Brazil

**Keywords:** respondent-driven sampling, electronic nicotine delivery systems, web-based surveys, online respondent-driven sampling, web-RDS, hard-to-reach populations

## Abstract

**Background:**

The marketing of electronic nicotine delivery systems (ENDSs) has been prohibited in Brazil since 2009, and their regular use is less prevalent than in countries where these devices are not banned. To monitor the presence of ENDSs, it is important to prevent the development of a new generation of nicotine-dependent individuals. However, traditional surveys are costly for accessing rare populations. Therefore, to reach ENDS users aged ≥15 years, we used the online version of the respondent-driven sampling method (web-RDS), a peer chain recruitment method for contacting hard-to-reach groups.

**Objective:**

This paper aims to provide information on the implementation of the first web-RDS study in Brazil to recruit ENDS users.

**Methods:**

This study was conducted in Rio de Janeiro, the second largest city in Brazil. After a formative phase using qualitative in-depth interviews, we selected the first participants (“seeds”) to complete an online quantitative questionnaire on the profile of their own ENDS use and the size of their contact network of ENDS users. Participants received 3 coupons to invite eligible peers. For participation and recruitment, each participant received a gift card worth approximately US $4. The target sample size was 300 ENDS users based on a conservative estimate and adjusted for design effect.

**Results:**

From August 2022 to May 2023, of the 12 seeds included, 508 attempts at access were recorded in the data collection system, of which 330 (65%) were eligible. Duplicate or ineligible attempts were identified and removed through automated and manual checks. Recruitment was initially slow due to the low monetary incentive, but it improved after the amount was increased. We found that 43.1% (75/174) of recruiters recruited only 1 eligible participant, 34.5% (60/174) recruited 2 eligible participants, and 22.4% (39/174) recruited 3 participants. Web-RDS was able to reach individuals in different areas of the city. Convergence was reached for target variables (ie, age and age at first use of electronic cigarettes). The median time to complete the questionnaire was 12 (IQR 8-17) minutes. Half (154/324, 47.5%) of the respondents reported that they knew up to 5 other ENDS users.

**Conclusions:**

The web-RDS methodology proved to be a feasible approach for accessing the population of ENDS users in Brazil. Incentives for participation and recruitment emerged as a determining factor in the data collection process. However, researchers needed to be aware of individuals attempting to circumvent the system by participating without being eligible or participating multiple times. Implications for optimizing web-RDS are discussed. On the basis of the method’s performance in this study, web-RDS shows potential to support future repeated data collection processes that could help monitor changes in the profiles of ENDS users over time, supporting the implementation of ongoing measures from Brazil’s National Tobacco Control Policy.

## Introduction

Electronic nicotine delivery systems (ENDSs), among which the most widely known are electronic cigarettes, have been used by the tobacco industry to replace (and possibly expand) the consumer base of nicotine-dependent individuals. This is because a global decline in cigarette consumption has been observed as a result of various tobacco control measures based on unequivocal evidence of the diseases and deaths caused by tobacco products [1,2].

When ENDSs were first introduced into the market, the tobacco industry promoted them on the grounds of harm reduction properties for people’s health, supplying nicotine safely to individuals during smoking cessation [3,4]. However, there is no evidence to support this claim because electronic cigarettes cause serious lung damage [5]. In addition, the odds of developing cardiovascular disease, stroke, and metabolic dysfunction are similar between users of conventional vs electronic cigarettes [6]. Moreover, several studies indicate that switching from conventional cigarettes to ENDSs does not lead to smoking cessation, as it merely satisfies nicotine dependence, now administered in a different form.

The sale of ENDSs has been prohibited in Brazil since 2009, and the ban was reinforced by a new 2024 resolution from the Brazilian Health Regulatory Agency [7]. Current use is relatively rare as a result. According to the latest available nationwide data from the 2019 National Health Survey, prevalence of current ENDS use among Brazilians aged ≥15 years was 0.6% [8]. The most recent data from the Surveillance System of Risk and Protective Factors for Chronic Diseases via Telephone Survey conducted among adults living in Brazilian state capitals showed a downward trend in point estimates for the proportion of current ENDS use (from 2.5% in 2020 to 2.1% in 2023) [9]. However, these data are neither informative nor generalizable to the vast network of municipalities that are not state capitals (more than 5500 municipalities home to approximately 80% of Brazil’s population, according to the 2022 census), for which recent trends are unknown.

ENDSs have been widely promoted through illegal online advertisements, mainly via social media and digital influencers, which mainly target young people and may encourage smoking initiation [10]. Not surprisingly, in the age group most affected by the tobacco industry’s marketing (15 to 24 years), prevalence of ever use of electronic cigarettes is significant (5.4%), and prevalence of current use is 2.4% [8].

Large nationwide surveys in Brazil such as the Brazilian National Health Survey and Surveillance System of Risk and Protective Factors for Chronic Diseases via Telephone Survey do not provide the necessary details to understand the patterns of ENDS use, consumer behaviors, motivations for use, and methods of access to these devices. In addition, population-based household or telephone surveys would require large numbers of participants to reach ENDS users, who represent less than 1% of the general population in Brazil and are not proportionally distributed according to the general population’s demographic profile.

Thus, specific methodologies are needed to access hard-to-reach or rare populations. The implementation of these methods is necessary to identify and quantify such populations and inform public policies and political and administrative decisions, such as those made by the Brazilian Health Regulatory Agency. One such methodology is respondent-driven sampling (RDS), initially developed to access more vulnerable populations at risk of key infections such as HIV and other sexually transmitted infections, and among injecting drug users [11]. However, RDS has since been applied to different contexts and groups, such as migrant populations, Indigenous people, drug users, female sex workers, and men who have sex with men [12].

In RDS, an initial group of participants, known as seeds, is purposely selected by the researchers. From there, each participant recruits a predetermined number of other eligible participants, and this process continues until the intended sample size is reached. This method can be viewed as asymptotically probabilistic [13] as it allows for the calculation of selection probabilities and sampling weights with a reasonable degree of approximation and acceptable error [14,15]. Estimation in RDS relies on participants’ self-reported personal network size, which reflects the number of eligible peers they know. This information is used to calculate each participant’s likelihood of selection and compute sampling weights, allowing for less biased population estimates. However, it cannot reach people who are not connected to contact networks, as they have zero probability of selection, and the accuracy of the probability of selection and its estimation depends on the structure of community geography and underlying contact networks [16]. For participation and recruitment, participants usually receive monetary incentives [12].

RDS traditionally uses face-to-face interviews. However, a new modality of RDS has been proposed more recently, where individuals are recruited by their peers but, rather than participating in person, they complete an online questionnaire. This modality has been called web-RDS [17]. It offers several advantages over traditional RDS, such as easier and anonymous access to participants in addition to enabling faster, less expensive, and more logistically efficient data collection [18]. The method naturally depends on the target population’s digital connectivity, which is especially high among Brazilian youth across all social classes, with only minor inequalities affecting low-income groups [19,20].

A review article identified 18 studies published from 2000 to 2019 that used web-RDS conducted in 8 countries [18]. Other more recent studies have also used this methodology [21-23]. Web-RDS has been used in these studies for population size estimation, health interventions, and the study of contact network characteristics (eg, mapping the spread of infections or diseases). However, the distribution of complex behaviors still lacks an appropriate methodology for surveys beyond experimental studies [24] and across various types of populations. There are currently no records of this methodology’s use in Brazil.

This paper aims to provide information on the implementation of a web-RDS study to recruit users of ENDSs. This is the first study in Brazil with this target population and methodology. We also aimed to gather insights to support the scalability of this methodology, with a view toward its simultaneous use in various sites across the country for comparison among different subgroups of ENDS users.

## Methods

### Study Design and Target Population

A cross-sectional study was conducted using web-RDS to collect data online, recruiting ENDS users in the city of Rio de Janeiro. This study was intentionally limited to 1 city as an implementation test for feasibility, recruitment dynamics, and system functionality before considering possible multicity expansion.

Eligibility criteria for participation in the study were having used ENDSs in the previous 12 months, age of ≥15 years, and residence in the city of Rio de Janeiro. This study considered the use of electronic cigarettes, heated tobacco, or vaporizers for tobacco consumption regardless of frequency of use in the 12 months before participating in the study.

### Formative Phase

A formative study using qualitative methodology was conducted, as research suggests that this can be a key factor for RDS studies’ ability to achieve their objectives [25]. The formative phase is important because it allows researchers to verify whether the target population is indeed connected in a way that enables chain recruitment. It also supports the selection of seeds (ideally well-connected, motivated, and heterogeneous individuals vis-à-vis socioeconomic status and location) to initiate the data collection process in RDS. It further facilitates the choice of the most effective primary and secondary incentives for online recruitment and can help refine the questionnaire [25].

Two pilot interviews were conducted to assess comprehensibility, acceptability, and feasibility based on implementation science criteria [26]. After these interviews, necessary adjustments were made to the scripts to improve the flow of the interviews. These 2 interviews were not included in the final set for analysis.

In-depth interviews were conducted from July 2022 to September 2022 with 14 individuals who met the eligibility criteria. These individuals were recruited using a combination of purposeful and snowball sampling. The researchers issued online invitations to key informants within their contact networks. These informants, in turn, passed the invitation on to potential participants or others in their networks who could recommend additional participants. Upon confirming the individuals’ interest in participating, the researchers checked their eligibility before conducting the interviews. The interviews followed a semistructured script with guiding questions divided into thematic sections: general sociodemographic and behavioral characteristics, experience with ENDSs, experience with traditional cigarettes, legislation, and the ENDS users’ contact networks. The qualitative interviews lasted 20 to 40 minutes and were recorded and transcribed in full for analysis.

### Sampling Plan

To determine the sample size for the quantitative phase, a conservative estimate of 50% was used for the sample calculation (as it maximizes variance and yields the largest required sample size, providing adequate precision across a range of possible true prevalence values), with an SE of 0.05. Considering a design effect of 2.5 [27], the minimum sample size was calculated as 250 individuals. To account for potential data losses, especially common in studies involving stigmatized and/or criminalized behaviors [28], the sample size was increased by 20%. Therefore, the target sample size for this study was 300 ENDS users.

### Seed Selection and Recruitment

Seeds were initially selected from individuals who participated in the qualitative phase of the study. The first 7 seeds were contacted in August 2022 and invited to complete an online questionnaire through a link provided via email. A dedicated email account was created for the study through which invitations were sent to the seeds. This email account was also used as the official communication channel for participants, including for inquiries.

During the data collection period, it became necessary to include additional seeds to reach the intended sample size and increase the network’s diversity, thereby minimizing the effects of homophily or the tendency for participants to recruit others with similar characteristics [13,15]. A total of 15 seeds were contacted, comprising individuals who participated in the formative phase or others they indicated. However, of these 15 seeds, 3 (20%) failed to complete the questionnaire. Thus, this study included 12 valid seeds distributed across the city of Rio de Janeiro as follows: 6 (50%) from the south zone, 4 (33.3%) from the north zone, 1 (8.3%) from the west zone, and 1 (8.3%) from the city center. There were 75% (9/12) men and 25% (3/12) women aged 21 to 48 years, with 25% (3/12) of the participants aged ≤24 years, 33.3% (4/12) aged 25 to 34 years, and 41.7% (5/12) aged ≥35 years. All reported having used ENDSs for more than 2 years. After completing the questionnaire, each seed was given 3 unique codes to invite eligible friends to participate.

### Data Collection System

Data collection was designed so that the questionnaire could be completed on a computer or cellphone, with the option to pause and resume completion later.

To begin the questionnaire, after reading and providing consent, participants had to enter their invitation code, email address, and date of birth. This information was used to verify duplicate participation. Participants then completed a self-administered online eligibility questionnaire. To address potential duplicate or fraudulent entries, especially in the context of an incentive-driven web survey, we implemented a multistep verification process combining automated and manual checks. First, the system automatically blocked repeated attempts using the same invitation code, email address, or date of birth. Second, manual validation was conducted to detect patterns consistent with the same individual attempting to participate multiple times, such as highly similar email structures (eg, inversions of first and last names and numerical variants of the same basic username) or different email addresses associated with an identical or implausibly similar date of birth. Attempts exhibiting any of these characteristics were classified as invalid and excluded from the sample for analysis. Additionally, cases in which different email addresses matched the recruiter’s own date of birth were removed as this indicated possible self-recruitment. Fraud detection methods cannot guarantee full elimination of invalid entries, but they substantially increase confidence that the data included in the analysis come from verified and legitimate participants.

Upon meeting the study inclusion criteria, participants were allowed to proceed to the formal study questionnaire, which contained modules with questions on sociodemographic characteristics and use of conventional cigarettes, electronic cigarettes, heated tobacco products, dry herb vaporizers, hookahs, other tobacco products, and alcohol. For ENDSs, the questions addressed use patterns and frequency, age of initiation, access methods, and motivations for use. Participants were also asked about their knowledge of legislation regarding ENDS advertising and use in public places. Additionally, the questionnaire included questions about the participants’ contact networks, as required by the RDS methodology, to assess their networks and allow for calculation of sampling weights and population estimates.

Three invitation codes were generated automatically at the end of the questionnaire. Coupons or codes were electronic, generated automatically by the system, and consisted of alphanumeric codes assigned randomly without reuse. These codes could be shared directly via WhatsApp or email by clicking on specific links or copied and sent manually through social media apps, for example. Codes had no expiration date but became inactive after use by an individual. In addition, the data collection system only remained active until the target sample size was reached, after which no additional enrollments could be made.

### Individual and Public Involvement

Upon completing the questionnaire, each participant received 3 new unique codes to invite friends to participate based on the eligibility criteria. Thus, participants also acted as recruiters, following the RDS methodology.

### Management of Invitations and Incentives

iFood gift cards were purchased to use as the primary incentive (for participation) and secondary incentive (for recruiting other participants). Initially, the vouchers were worth BRL 10 each (slightly less than US $2). However, this amount had to be increased to BRL 20 (slightly less than US $4 at the time) to encourage recruitment. This adjustment was made after seeds reported that the low amount of the incentive was the main reason for the lack of recruitment and participation by invitees.

Email messages containing primary and secondary incentive gift card information were sent manually by the study coordinator to each participant using the email address provided by the participant when completing the questionnaire. Email messages were also sent to participants to encourage them to invite friends to join when their codes had not been used after an extended period. These email messages also informed participants that the gift card could not be provided if evidence of duplicate participation was detected manually by the research team. They were also sent in response to inquiries. Approximately 700 email messages were sent during the study period.

### Contact Network Size

The size of each participant’s contact network was measured using the following 2 questions: “How many people do you know who have used ENDS in the last 12 months and live in the city of Rio de Janeiro?” and “How many of these people are 15 years or older?” (the latter being the operative network size for weighting). This information’s validity was important for estimating the probability of inclusion in the study and for obtaining reliable weighted estimates [29]. Therefore, the consistency of these data was verified, and deterministic imputation was applied for implausible responses to the question defining the probability of inclusion in the study. When the reported network size in the second question was zero or smaller than the number of individuals connected to the respondent in the recruitment tree (ie, including both the recruiter and the recruits [13 cases]), we imputed the value provided in the first question. Conversely, when the first question also yielded 0 or an implausibly small value relative to the observed recruitment ties (7 cases), the network size was replaced by the mean of the valid network sizes observed in the sample. This approach follows conservative recommendations in the RDS literature by avoiding artificially small degrees, which would otherwise generate disproportionately large sampling weights. Imputation was applied to approximately 6% of the responses. The distribution of the contact network size variable was presented in box plots and histograms.

### Analyses

The analyses were conducted using the R programming language (version 4.3.1; R Foundation for Statistical Computing) [30]. Because our analyses in this implementation study were limited to unweighted descriptive summaries of the obtained sample, no RDS estimator was applied; population inference will be addressed in future analyses [31]. Convergence was assessed visually using cumulative sample proportion plots for selected target variables.

### Ethical Considerations

This study was approved by the institutional review board of the Brazilian National Cancer Institute (33280320.3.0000.5274). Consent to participate in the qualitative interviews was obtained from participants and recorded at the beginning of each interview after they were informed about the study’s objectives, methods, risks, and benefits. Similarly, before completing the questionnaire in the quantitative phase, individuals were required to consent to participate by clicking on a specific field in the online questionnaire. As described in the Management of Invitations and Incentives section, participants received a primary incentive for participation (a BRL 20 [US $4] gift card) and a secondary incentive for each successful peer recruitment (a BRL 20 [US $4] gift card, limited to 3 recruits, for a maximum total of BRL 60 [US $12]). To ensure privacy and confidentiality, only minimal personal information was collected, solely to prevent duplicate responses and to enable incentive distribution. All data were stored in secure databases, and participants’ personal information was not shared with third parties and was accessible only to the study coordinator.

## Results

**Figure 1:**
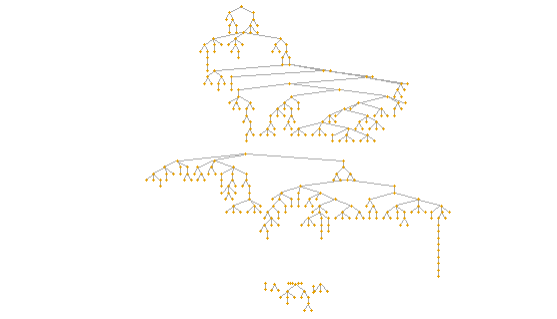
Recruitment tree of electronic nicotine delivery system users participating in the study (Rio de Janeiro, 2022-2023).

During data collection (August 2022 to May 2023), 508 attempts at access were recorded in the online system for participation in the study. However, 30.7% (n=156) of these were deemed ineligible (including manually identified duplicates, ie, people attempting to respond more than once while bypassing the automatic filter system). An additional 4.3% (n=22) of the participants failed to complete the questionnaire. Thus, the final study sample consisted of 330 individuals, with recruitment chains reaching a maximum depth of 21 waves (Figure 1); 5 seeds did not recruit any participants.

Database consistency analysis showed that 1.8% (6/330) of the individuals, although reporting previous use of ENDSs in the eligibility questionnaire, failed to provide valid or consistent information on the use of electronic cigarettes, heated tobacco cigarettes, or vaporizers for tobacco use. Thus, they were excluded from the analyses presented in this paper and future analyses (but will be retained for generating the recruitment network).

The sample consisted of 38.3% (124/324) individuals aged 16 to 24 years and 41.7% (135/324) individuals aged 25 to 34 years. Female individuals accounted for 43.8% (142/324) of the sample, and male individuals accounted for 56.2% (182/324). Most respondents (263/324, 81.2%) reported ever having smoked conventional cigarettes, and 68.1% (179/263) of these respondents were current smokers. Nearly the entire sample (317/324, 97.8%) reported having ever used electronic cigarettes, whereas experimentation with heated tobacco and vaporizers was reported by approximately one-third of the participants (109/324, 33.6% and 106/324, 32.7%, respectively; results not shown in table format). These descriptive characteristics provide a basic overview of the individuals captured through the recruitment chains using web-RDS.

**Figure 2:**
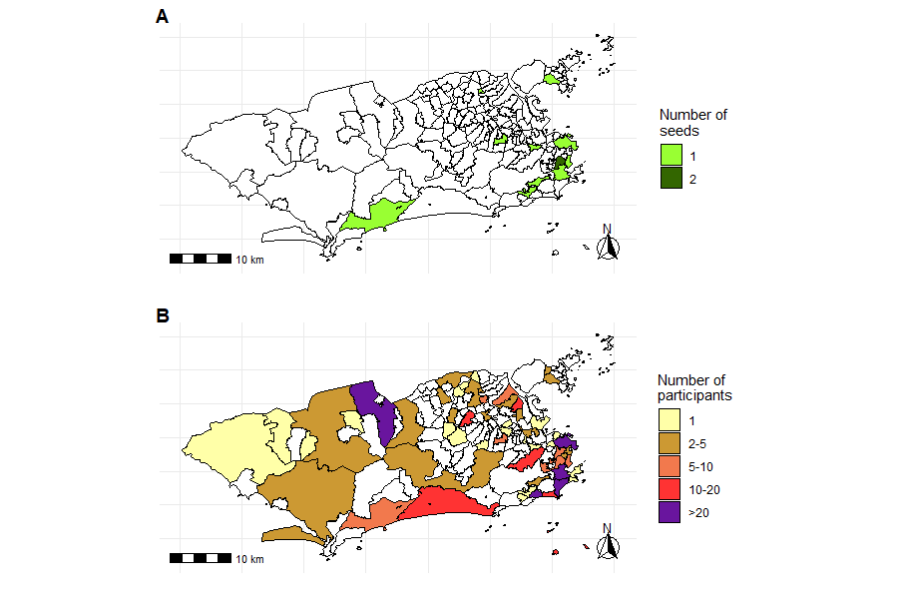
Neighborhood of residence of seeds (A) and total sample (B) of electronic nicotine delivery system users participating in the study (Rio de Janeiro, 2022-2023).

The sample obtained using web-RDS included individuals in different areas of Rio de Janeiro, although the seeds were limited to a small number of neighborhoods in a city with more than 6 million inhabitants (Figure 2).

**Figure 3:**
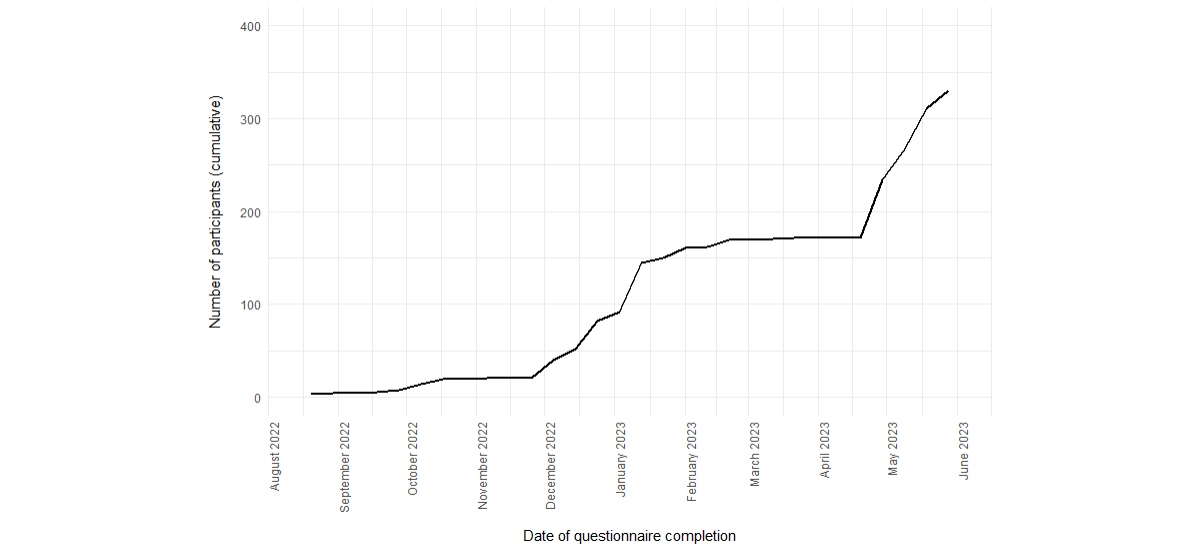
Number of electronic nicotine delivery system users participating in the study by date (Rio de Janeiro, 2022-2023).

Figure 3 shows the distribution of participants by date of questionnaire completion. The initial weeks had the fewest participants, as this was when data collection began with the seeds’ participation in August 2022 and September 2022. There was a delay in participation by the seeds’ invitees, who were reluctant to participate because they considered the gift card value too low (BRL 10 [US $2] at the time). Therefore, starting in October 2022, the amount was increased to BRL 20 (US $4). None of the seeds generated valid recruits before the increase in the incentive value. Following this change, and with email messages sent to encourage participation and peer recruitment, the chain recruitment gained the necessary momentum starting in November 2022.

There was a plateau in participation from January 2023 to April 2023 (Figure 3), indicating that few interviews were completed during this period. This may have been due to a bureaucratic issue in early January 2023 that delayed the purchase of new gift cards, which could not be completed within the 7-day time frame promised to participants in the informed consent form and the invitations. Given the restrictions on the number of gift cards, we decided to maintain the primary incentive, whereas the secondary incentive was resumed in the latter half of February 2023. Even with email messages encouraging participants to recruit, there was no proactive and swift response as before. Thus, to reach the target sample size, and following the suspension of restrictions on gift cards, new seeds were included in late April 2023, leading to a gradual increase in the sample size at a rate consistent with that of the previous period.

Importantly, although each participant received a maximum of 3 invitation codes, in the final valid sample, we found that 43.1% (75/174) of recruiters enrolled only 1 eligible participant, 34.5% (60/174) recruited 2 eligible participants, and 22.4% (39/174) successfully recruited 3 participants (data not shown in table format). Considering the 508 individuals reached by the study (regardless of potential duplications), we found that approximately half (113/218, 51.8%) used all 3 invitations (data not shown in table format).

**Figure 4:**
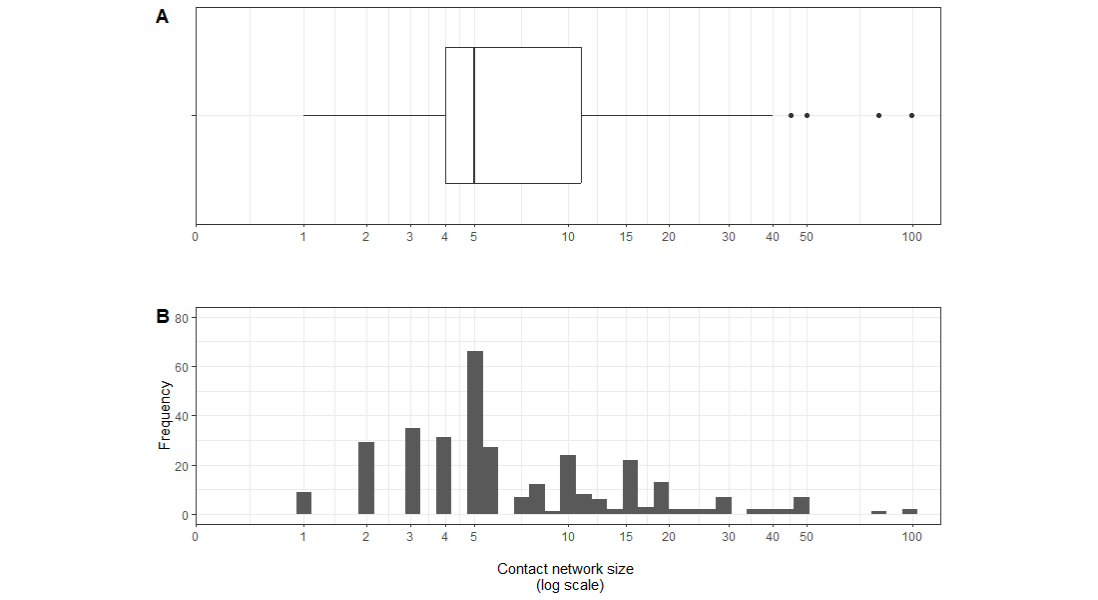
Boxplot (A) and histogram (B) of contact network size of electronic nicotine delivery system users participating in the study (Rio de Janeiro, 2022-2023).

Figure 4 shows the distribution of contact network size among study participants (ie, the number of ENDS users aged ≥15 years residing in Rio de Janeiro that the respondents reported knowing). Half (154/324, 47.5%) of the respondents reported that they knew up to 5 other ENDS users (this number corresponds to the mode of the self-reported contact networks). Only 25.6% (83/324) of the participants reported having a contact network with more than 10 eligible individuals (the maximum reported number was 99).

The median time for completion of the questionnaire was 12 (IQR 8-17) minutes.

**Figure 5:**
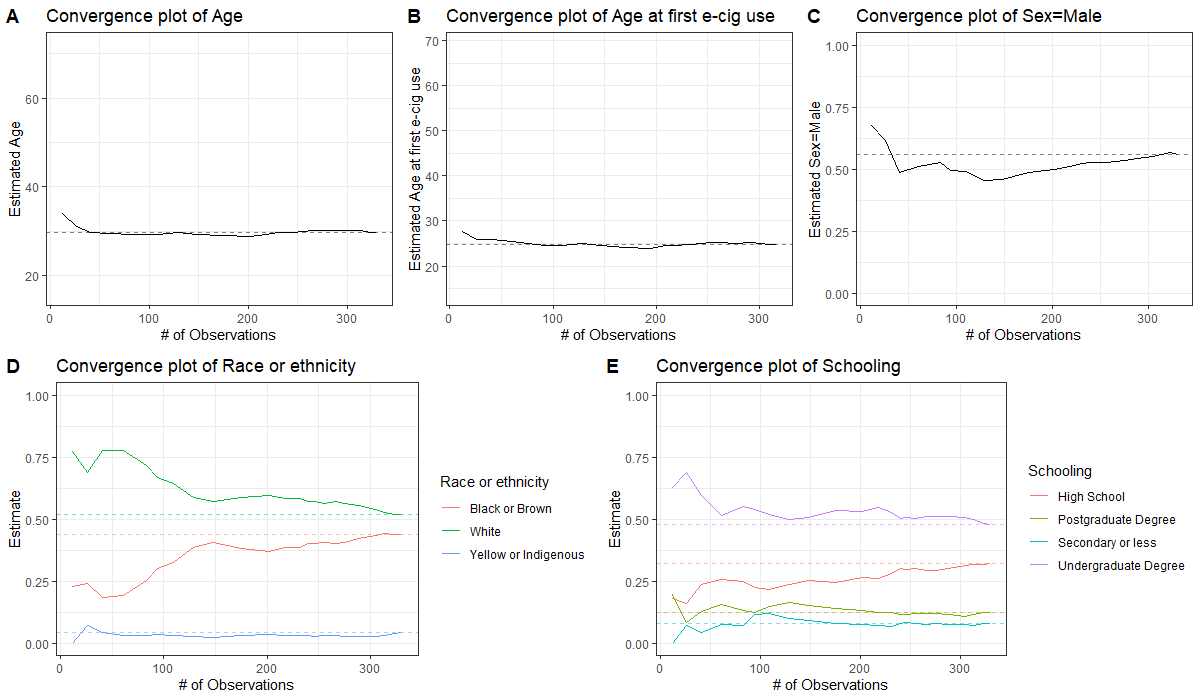
Convergence plots for selected variables of electronic nicotine delivery system users participating in the study (Rio de Janeiro, 2022-2023).

As shown in Figure 5, different participant characteristics exhibited distinct convergence trajectories, sometimes reinforcing the presence of homophily, other times showing chain referral processes that followed other patterns. Convergence was reached for target variables (ie, age and age at first use of electronic cigarettes).

## Discussion

### Principal Findings

Web-RDS proved to be a feasible method for accessing the population of ENDS users, reaching participants in nearly all areas of the city of Rio de Janeiro. Although RDS has been used in several other studies in Brazil, ours was the first to use web-RDS and the first to use this methodology to access ENDS users. Our results are important for implementing web-RDS to ensure more effective data collection, as outlined in the following paragraphs.

Incentives for participation and recruitment emerged as a determining factor in the data collection process, consistent with findings in the international literature [18]. Recruitment chains only initiated after the primary incentive was increased, indicating that early recruitment failure was primarily related to insufficient incentive rather than to lack of network connectivity. Therefore, ensuring that there is no shortage of incentives and that they are provided as quickly as possible after completing the questionnaire can improve the effectiveness of data collection given the motivation to participate and to invite others [32]. In many settings (but not in Brazil), the legislation allows for cash compensation and incentives for participation in such studies; however, this requires a logistics system that may be unavailable in many countries. Double incentives are an intrinsic feature of studies using RDS [11]. Although several studies have attempted to quantify the influence of referral bias associated with illicit procedures or strategic participation, such efforts face a fundamental limitation: the impossibility of fully and consistently quantifying human decision-making under ethical and contextual constraints [33]. While strategic decisions may improve respondent-driven studies, they do not constitute rigorous conceptual or mathematical solutions for disentangling the combined influence of all underlying factors.

Researchers should be aware of attempts by individuals to circumvent the system by participating without being eligible or participating multiple times. This disadvantage of offering incentives for participation has also been observed in other studies with various populations [18], but it can be mitigated by using different methods, both automated and manual, to check for duplicate participation. A verification system such as one using genetic or evolutionary algorithms to track each web connection might be feasible [34]; however, it would jeopardize the study’s confidentiality and violate its ethical assumptions. This concern is not exclusive to web-RDS studies, but it is a recurring issue in online research in general, particularly when pecuniary compensation is involved [35]. In our study, whenever fraudulent attempts were detected, respondents received an email message explaining that they were only allowed to participate once and that they would not be compensated (their invitations were also blocked). Such strategies have been used in other studies [21] and can deter attempts at multiple participation.

The use of web-RDS to collect large or multicenter samples requires even more automated systems, but this does not eliminate the need for manual validation. Fraud is still possible even with more advanced systems or the use of mobile phone numbers as participant identifiers [21]. Using different strategies helps minimize this problem while preserving the ethical research principles. The number of invitations distributed per participant was sufficient in our study, and there was no need for more than 3, as less than one-fourth of the participants (39/174, 22.4%) successfully recruited 3 eligible participants. In addition, half (154/324, 47.5%) of the users reported networks of up to 5 individuals. However, when a participant recruited an ineligible individual, the code became invalid and could not be reused to invite others. Approximately one-third of the invitations (178/508, 35.0%) were used by ineligible individuals, highlighting the importance of reinforcing the eligibility criteria with participants when recruiting their peers.

The upper limit of contact network size in our study is consistent with Dunbar’s number, which theoretically represents the cognitive limit on the number of stable relationships a person can maintain [36]. However, this point deserves special attention due to the ongoing debate regarding the validity of Dunbar’s number [37]. Respondents in our study tended to report their network size as a multiple of 5 (for networks larger than 5 individuals), as reported in other studies [38]. Therefore, the use of such strategies for estimating weights should be addressed, as there is a risk of bias, particularly when accessing consecutive samples. While larger samples would reduce uncertainties regarding the size of contact networks (degree) [29], they would not eliminate them given the persistent challenge of bottlenecks in any nomination network. There are strategies to minimize the negative impact of bottlenecks on recruitment processes, but bottlenecks cannot be entirely ignored [39]. Additionally, several methods for correcting or weighting samples obtained through RDS have been discussed [40,41], even the nonuse of weights for regression models [42].

Homophily is a permanent challenge for chain referral processes to recruit diverse samples rather than being trapped by “clonal dominance.” The latter is quite common in biology [43] and has been extrapolated to social processes, with observable consequences [44] (ie, biasing the recruiting process). The seminal paper by Goel and Salganik [45] shows that RDS can follow a more complex process than originally conceived (as a first-order Markov process). Viewing RDS as a Markov chain Monte Carlo not only helps us understand “the effects of community structure and the recruitment procedure on the variance of RDS estimates,” as stated by the authors, but also explains our study’s findings. Homophily exists but is not the norm or an inevitable finding of RDS studies. The pervasive idea of “six degrees of separation” (frequently understood as a metaphor rather than a sound mathematical framework or a concrete empirical finding) suggests that most individuals can be reached within 6 intermediaries, which inspires the use of chain referral methods to access hidden populations [46]. However, this theory refers to network reach, not the specific statistical condition of sample equilibrium required for unbiased population estimates in RDS analysis.

### Limitations

This study used email to communicate with participants (for motivation and to distribute incentives); however, through contact with the “seeds,” we found that younger participants used email less frequently and preferred to be contacted via WhatsApp. Other studies have used cellphone numbers both as identifiers and to contact participants [21]; however, the automated use of WhatsApp is more complex and should be evaluated in light of the study’s objectives [22].

Although this study used automated and manual verification procedures, additional antifraud tools (eg, one-time passwords sent to phone numbers, geolocation checks, or IP-based detection) may further reduce duplicate attempts at participation, but they were not used in this study. These methods involve trade-offs related to privacy, cost, and system complexity. Previous evidence [47] has shown that no single measure is fully effective in incentive-based online surveys and that in-depth verification remains essential.

Another limitation of the method is that it obviously cannot reach populations that lack internet access. However, this does not appear to be an issue when applying web-RDS to users of electronic smoking devices, as studies in Brazil indicate that this group enjoys high income and schooling levels [48]. According to the Brazilian census bureau, the entire educated population in Brazil has internet access [20]. If the use of ENDSs spreads to less privileged population groups, as is often the case with technological innovations [49], digital exclusion could introduce selection biases. It is also true that internet access is expanding across different population strata in Brazil [20]. Importantly, this study was only performed in the city of Rio de Janeiro, so the findings should not be generalized to the entire population of Brazilian ENDS users. However, the web-RDS procedures tested in this study provide insights into the feasibility of broader recruitment of ENDS users in Brazil.

The processes and outcomes in RDS studies have still not achieved the full application of implementation science concepts and methods. This limitation should be addressed by future studies inspired by new approaches to RDS methods and implementation science [26].

### Conclusions

The web-RDS methodology proved capable of reaching ENDS users in different neighborhoods of Brazil’s second largest city within a short period and at a relatively low cost. These characteristics could allow for the replication of the study at short intervals and on a larger scale for recruiting ENDS users across Brazil as a whole. Thus, web-RDS would enable monitoring the profile of ENDS users and potential changes over time, as well as methods for acquiring such products in different social and geographic contexts, thereby supporting the implementation of ongoing measures from Brazil’s National Tobacco Control Policy.

## Abbreviations

ENDS: electronic nicotine delivery system
RDS: respondent-driven sampling
Web-RDS: web-based respondent-driven sampling

## Data Availability

The datasets generated or analyzed during this study are not publicly available due to anonymity and confidentiality but are available from the corresponding author on reasonable request.
